# Epigenetics and gestational diabetes: a review of epigenetic epidemiology studies and their use to explore epigenetic mediation and improve prediction

**DOI:** 10.1007/s00125-019-05011-8

**Published:** 2019-10-17

**Authors:** Hannah R. Elliott, Gemma C. Sharp, Caroline L. Relton, Deborah A. Lawlor

**Affiliations:** 1 MRC Integrative Epidemiology Unit at the University of Bristol, University of Bristol, Oakfield House, Oakfield Grove, Bristol, BS8 2BN UK; 2 Population Health Sciences, Bristol Medical School, University of Bristol, Bristol, UK; 3 Bristol Dental School, University of Bristol, Bristol, UK; 4 Bristol NIHR Biomedical Research Centre, University of Bristol, Bristol, UK

**Keywords:** Epidemiology, Epigenetics, Gestational diabetes, Mediation prediction, Pregnancy, Review

## Abstract

Epigenetics encapsulates a group of molecular mechanisms including DNA methylation, histone modification and microRNAs (miRNAs). Gestational diabetes (GDM) increases the risk of adverse perinatal outcomes and is associated with future offspring risk of obesity and type 2 diabetes. It has been hypothesised that epigenetic mechanisms mediate an effect of GDM on offspring adiposity and type 2 diabetes and this could provide a modifiable mechanism to reduce type 2 diabetes in the next generation. Evidence for this hypothesis is lacking. Epigenetic epidemiology could also contribute to reducing type 2 diabetes by identifying biomarkers that accurately predict risk of GDM and its associated future adverse outcomes. We reviewed published human studies that explored associations between any of maternal GDM, type 2 diabetes, gestational fasting or post-load glucose and any epigenetic marker (DNA methylation, histone modification or miRNA). Of the 81 relevant studies we identified, most focused on the potential role of epigenetic mechanisms in mediating intrauterine effects of GDM on offspring outcomes. Studies were small (median total number of participants 58; median number of GDM cases 27) and most did not attempt replication. The most common epigenetic measure analysed was DNA methylation. Most studies that aimed to explore epigenetic mediation examined associations of in utero exposure to GDM with offspring cord or infant blood/placenta DNA methylation. Exploration of any causal effect, or effect on downstream offspring outcomes, was lacking. There is a need for more robust methods to explore the role of epigenetic mechanisms as possible mediators of effects of exposure to GDM on future risk of obesity and type 2 diabetes. Research to identify epigenetic biomarkers to improve identification of women at risk of GDM and its associated adverse (maternal and offspring) outcomes is currently rare but could contribute to future tools for accurate risk stratification.

**Electronic supplementary material:**

The online version of this article (10.1007/s00125-019-05011-8) contains peer-reviewed but unedited supplementary material including a slideset of the figures for download, which is available to authorised users.

## Epigenetic epidemiology and its use in gestational diabetes research

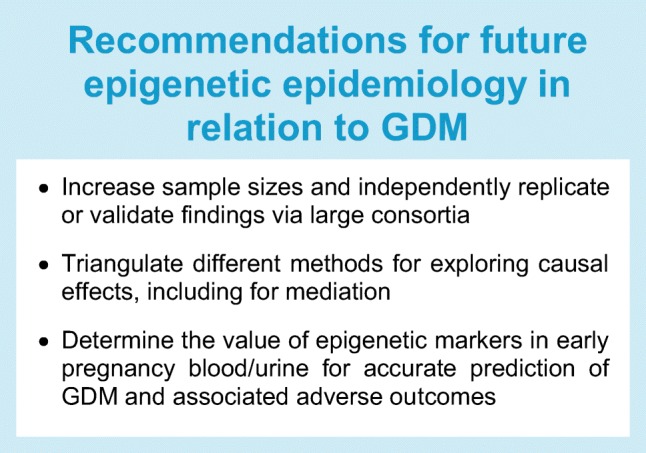



Epigenetics encapsulates a group of molecular mechanisms, including DNA methylation, histone modification and microRNAs (miRNAs), which can influence gene expression and variation in both cellular and whole-organism phenotype. An increasing number of clinical applications are emerging that use data generated in the field of epigenetic epidemiology. These include studies increasing our understanding of mechanistic pathways culminating in adverse health outcomes across the life course [1] as well as the use of epigenetic biomarkers as informative biomarkers in diagnosis, risk prediction and prognosis [2, 3].

In this review we describe the potential of epigenetic research: (1) to improve understanding of the causal paths between in utero exposure to gestational diabetes (GDM) or pregnancy hyperglycaemia and offspring adiposity and type 2 diabetes (type 2 diabetes) risk; and (2) as biomarkers for increasing the accuracy of predicting GDM risk and its associated adverse (maternal and offspring) outcomes. We then review and summarise current published human studies on the epigenetic epidemiology of GDM with a focus on these two areas of research.

### Epigenetic mediation of in utero exposure to GDM on offspring health

**Fig. 1:**
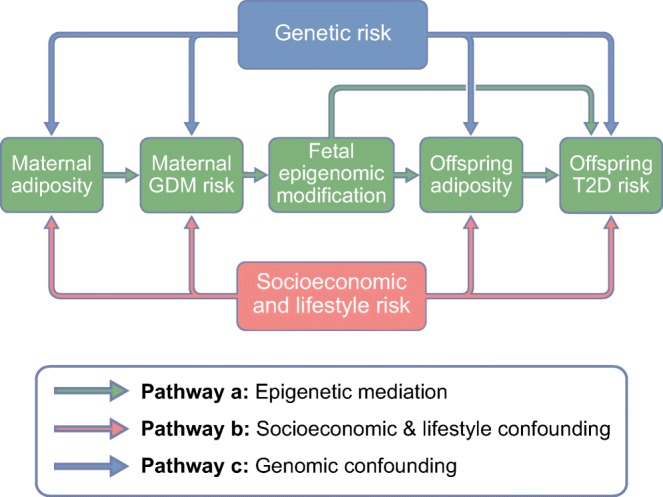
Summary of pathways that produce an intergenerational association between GDM and offspring type 2 diabetes (T2D). This figure is available as part of a downloadable slideset

Normal pregnancy is associated with insulin resistance, particularly from the second trimester, similar to that found in type 2 diabetes [4–6]. These changes facilitate transport of glucose across the placenta to ensure normal fetal growth and development [4–6]. If maternal gestational insulin resistance becomes too pronounced then maternal GDM may be diagnosed. Traditionally, GDM has been defined as any hyperglycaemia that is first identified during pregnancy, including existing undiagnosed diabetes/hyperglycaemia. Whilst early pregnancy tests are increasingly used to identify and treat women with existing hyperglycaemia [7], this is not universal and any impact of ‘GDM’ on epigenetic mechanisms or adverse outcomes may be due to existing hyperglycaemia or pregnancy-induced insulin resistance. GDM is associated with adverse perinatal [6] and longer-term offspring outcomes, including higher adiposity and adverse cardiometabolic risk factors such as higher circulating glucose and insulin [6, 8–10]. The latter may be due to developmental overnutrition and it has been hypothesised that epigenetic dysregulation is one mechanism underlying this association. However, other mechanisms could explain these associations, including shared familial socioeconomic, lifestyle and genetic factors [11–13] (Fig. 1, pathways b and c).

Mediation is concerned with causal effects but it is commonly explored through conventional multivariable regression using the method suggested by Baron and Kenny more than 30 years ago [14], without exploring the assumptions specified by those authors [15]. The challenges of researching causal molecular mediation, together with suggestions for novel appropriate approaches, have recently been described [15]. The path between maternal GDM and future offspring type 2 diabetes risk could be mediated by multiple mechanisms. For epigenetic mechanisms to mediate a hypothesised developmental origins path between in utero exposure to GDM and future offspring type 2 diabetes risk (Fig. 1, pathway a), evidence for all three of the following causal effects are required: (1) effect of GDM on future offspring type 2 diabetes; (2) effect of GDM on some epigenetic mechanism in relevant tissues; and (3) effect of those epigenetic mechanisms on future offspring type 2 diabetes. Determining such causal effects requires robust replication of associations and triangulation [16] of two or more different methods with different sources of bias for assessing causality, such as Mendelian randomisation (MR), [15], parental negative control studies [17], matched within-sibship designs [10] and cross cohort comparisons [16]. Within-sibship analyses provide some evidence for a causal effect of GDM on greater offspring BMI [8, 10]. In epigenetic epidemiology, a paternal negative control study found that most of the epigenome-wide associations of maternal early pregnancy BMI with cord-blood DNA methylation were similar to those of paternal BMI, suggesting the maternal associations were unlikely to be causal [17]. The extent to which such methods have been used to explore epigenetic mediating mechanisms between in utero exposure to GDM and offspring outcomes is one subject of this review.

Given the systems and tissues potentially involved in this hypothesised epigenetic mediating path, any research should ideally explore epigenetic mechanisms in offspring blood (including cord blood), placenta, pancreas, liver, muscle and adipose tissue. Access to blood and placental tissue should be feasible as an increasing number of birth cohorts collect cord blood or infant blood from screening blood spots, as well as placental tissue [18–22]. However, taking fat, muscle, pancreas and liver biopsies is unlikely to be feasible and ethical except in clinical cohorts where there is a clinical need. In silico reference data, such as that available in resources like the Genotype-Tissue Expression (GTEx) project [23], may be valuable for information on differential epigenetic phenomena in these tissues but those data are likely to come from small, select and usually adult populations.

### Epigenetics as biomarkers for diagnosis and risk prediction in relation to GDM

Current guidelines for screening and diagnosing GDM vary between countries and institutions. Universal OGTT of all pregnant women is rare and the benefit of doing this is debated [7, 24, 25]. The practice of early pregnancy risk factor screening to identify those at most risk of GDM (to enable selection for a later diagnostic OGTT) does not appear effective [24, 26, 27]. Other early pregnancy screening approaches, such as glucose challenge tests, HbA_1c_ and random or fasting glucose measurements, can be useful in identifying women with undiagnosed type 2 diabetes [7] but do not seem to be useful in identifying women with GDM or predicting associated adverse outcomes [24, 28]. A definitive diagnosis of GDM is made with an OGTT at around 26–28 weeks of gestation. However, emerging evidence shows that fetal growth trajectories already differ in those whose mothers are subsequently diagnosed with GDM compared with those whose mothers are not, from at least 12 weeks of gestation [29, 30]. Thus, there is a need for biomarkers that are measured on samples collected in early pregnancy that accurately predict GDM and its associated adverse perinatal and later (offspring and maternal) outcomes. These could indicate which women would benefit from early interventions (lifestyle or pharmaceutical) to reduce risk, including future risk of obesity and type 2 diabetes in mothers and offspring.

Unlike the use of epigenetic measures to explore mediation, their use as biomarkers does not require them to be causally related to the outcome they are predicting [31]. Causal methods and tissue specificity are therefore not the focus in epigenetic biomarker research. What is required is to show that epigenetic biomarkers that can be readily assessed in blood or urine (which are routinely collected at antenatal visits) improve the discrimination and calibration of current risk prediction tools. It is also important that prediction tools developed in one study are validated in independent studies.

## A review of current published literature

**Fig. 2:**
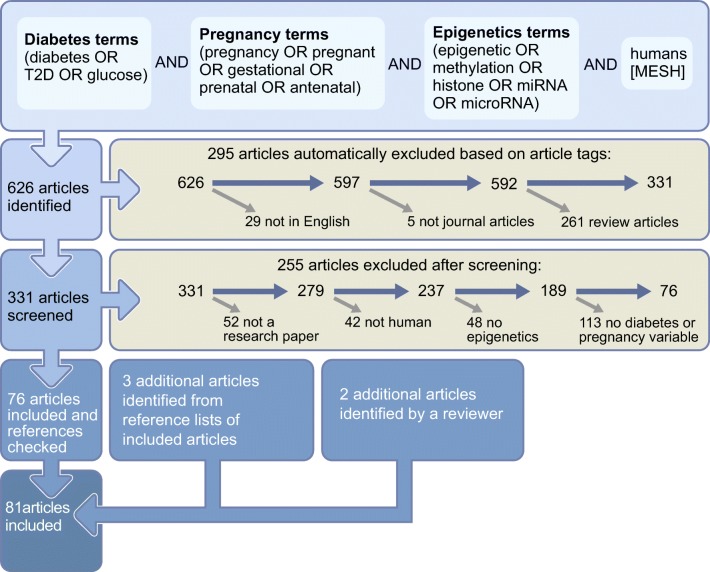
An overview of the PubMed search strategy to identify studies of interest. This figure is available as part of a downloadable slideset

**Fig. 3:**
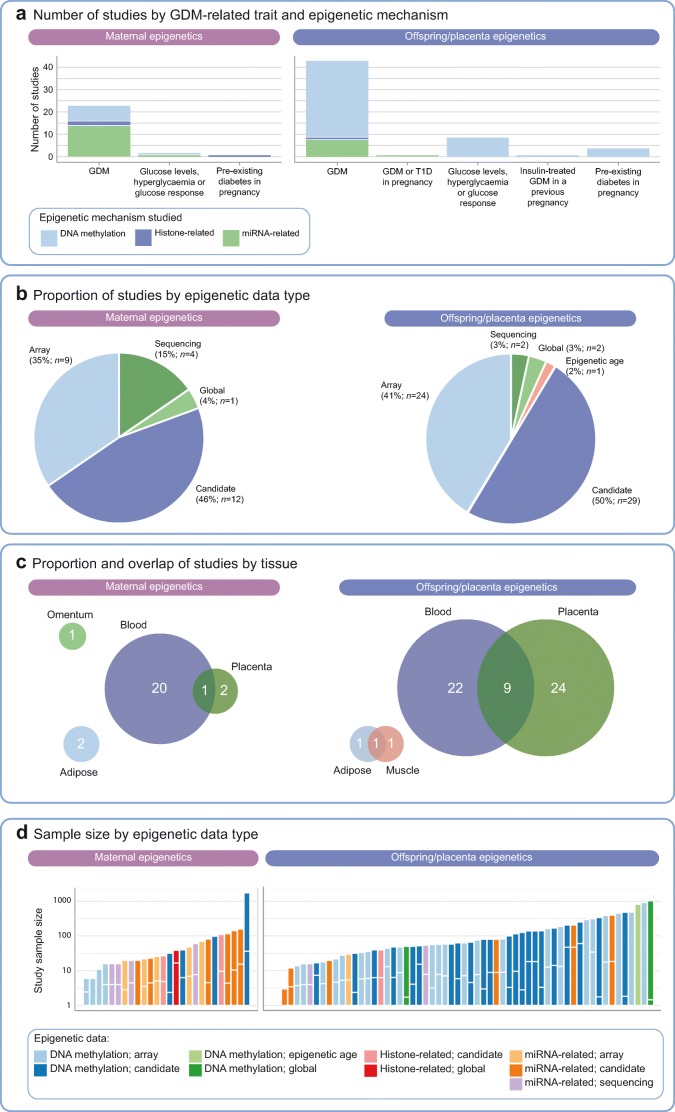
Summary of human epigenetic studies related to GDM or hyperglycaemia in pregnancy. In (**c**) the size of the circles reflects the number of studies in each tissue. The numbers of studies (given within the circles) total 84 (rather than 81) because three studies contributed to both of the broad areas and are depicted twice in this figure. In (**d**), for case–control studies, the length of the vertical bar below the white horizontal line shows the proportion of GDM cases, relative to the total length of the bar. T1D, type 1 diabetes. This figure is available as part of a downloadable slideset

We searched PubMed for any pregnancy-related studies that explored associations between any of maternal GDM, type 2 diabetes, gestational fasting or post-load glucose and any epigenetic marker (DNA methylation, histone modification or miRNA) (Fig. 2). We did not restrict our search solely to studies of GDM because of the varying methods that were likely to have been used to diagnose GDM in different studies and because existing hyperglycaemia or pregnancy-related insulin resistance may have influenced both short-term and long-term outcomes. We included any study, whatever its aim and whether it hypothesised epigenetic variation that preceded the diabetes-related outcomes or vice versa. Cross-sectional, case–control or cohort designs and global, epigenome-wide and candidate gene studies were all included. We restricted studies to those conducted in humans and written in English. We extracted key data from each study with the aim that this would provide important information on what is currently available in the published literature and how these studies might contribute to the two different potential uses of epigenetic epidemiology in GDM. We did not extract results or assess risk of bias in each study as this was considered beyond the scope of this paper. Similarly, we did not attempt to synthesise or pool results from different studies. We do, however, provide references from all studies and the data we extracted from them in electronic supplementary material (ESM) Table 2 (summarised in Fig. 3).

There were two main themes of research effort identified from our literature search: (1) studies of associations of GDM with offspring and/or placenta epigenetics (*n* = 55 studies), which were primarily concerned with epigenetic mediation of in utero exposure to hyperglycaemia on offspring subsequent health; and (2) studies of maternal epigenetics (*n* = 23 studies), which were mostly concerned with the role of epigenetics in the aetiology of GDM or its progression to type 2 diabetes. Three articles spanned both of these themes, so the total numbers of studies contributing to offspring/placenta and maternal epigenetics were 58 and 26, respectively, and the denominator used when we consider study themes (rather than individual papers) was 84 (Fig. 3).

Fifty-eight of the 84 studies (69%) explored associations of GDM, glucose levels/response or pre-existing maternal diabetes in pregnancy with offspring and/or placenta epigenetics (Fig. 3a). The most commonly studied epigenetic mechanism in these studies was DNA methylation (48/58; 83%). There were ten studies of offspring miRNA and one study of histones (Fig. 3a). There were 25 studies of offspring tissues (22 in blood, one in adipose, one in adipose and muscle, one in skeletal muscle), 24 studies of placenta and nine studies of both offspring blood and placenta (Fig. 3c). In most of these studies, the hypothesis or background rationale was that epigenetic mechanisms mediate any effect of GDM/glucose traits on offspring outcomes. However, these studies mainly presented associations of GDM (or a related exposure) with offspring/placenta methylation and exploration of any causal effect or effect on downstream offspring outcomes was lacking. Five of the studies conducted mediation analysis. In one study, two-step MR provided some evidence for differential DNA methylation levels near the leptin gene (*LEP*) mediating the effect of maternal fasting glucose on neonatal leptin levels [32]. This study was conducted in just 485 mother–offspring pairs and both offspring DNA methylation and leptin were measured in cord blood. In a study of 835 mother–offspring pairs, evidence from structural equation modelling suggested that GDM mediated an effect of obesity on fetal-side placental DNA methylation of the *LEP* promotor region [33]. Three further studies using the Baron and Kenny method reported evidence that DNA methylation might mediate the following effects: (1) the effect of in utero exposure to GDM on childhood cardiometabolic traits (specifically, differential methylation around *VCAM-1* [also known as *VCAM1*]) [34]; (2) the effect of maternal hyperglycaemia on offspring leptin levels at birth [35]; and (3) the effect of gestational type 2 diabetes on type 2 diabetes risk in offspring [36]. Of these five mediation analyses, one attempted to replicate findings in an independent study. Overall, just under half of the studies (27/58; 47%) attempted replication of findings in an independent cohort or conducted in vitro assays to support the main study findings, although at least 12 of the 58 studies (21%) noted the need for additional replication or validation (see ESM Table 2).

The second predominant theme of research effort was in identifying associations between maternal epigenetics and GDM or glucose levels/response in pregnancy (26/84 studies; 31%; Fig. 3a). Most of these studies aimed to explore the aetiology of GDM and/or the progression to type 2 diabetes. There were 20 studies of blood, two of the maternal side of the placenta, two of maternal adipose tissue, one of omentum and one of both blood and the maternal side of the placenta (Fig. 3c). Most studies of maternal epigenetics evaluated miRNA expression (15/26; 58%; Fig. 3a). Four studies explored prediction of GDM risk, using area under the receiver operating curve (AUROC) to test predictive discrimination and two of these four studies attempted to validate or replicate their findings. However, it was unclear whether the miRNAs identified from these studies were predictive of disease independently of known (clinical) predictors, or were more accurate than these known predictors, as these comparisons were not made.

Overall, among the 26 studies that examined maternal epigenetics as causal risk factors for (or predictors of) GDM, only ten (38%) attempted validation or replication of their results or included in vitro assays to support the main study findings. At least five of the 26 studies (19%) noted that additional replication or validation of study findings were needed (see ESM Table 2).

Across both research themes, studies were split evenly between candidate-gene- and array-based approaches, with a small proportion of studies assessing global or other measures (Fig. 3b). Sample sizes were small (median *n* = 58, median *n* cases = 27) and there was no obvious pattern of association between sample size and studied epigenetic mechanism or data type (Fig. 3d).

## Discussion: conclusions and future research

Our review shows that there is a substantial body of epigenetic epidemiology research in relation to GDM. Most of this articulates an interest in the possible mediation by epigenetic phenomena of a possible causal effect of maternal GDM on offspring future health including future risk of obesity and type 2 diabetes. Future research in this area should attempt to replicate findings, expand the range of causal analysis approaches applied to this question and, where possible, triangulate across these to explore whether epigenetic mechanisms that may be influenced by GDM relate to future adverse offspring outcomes.

Seeking replication and exploring causality through other methods such as negative control paternal studies and MR require large samples sizes and necessitate collaboration across studies. Thus, endeavours such as the Pregnancy And Childhood Epigenetics (PACE) consortium [37] are important for taking this research forward. Any study that has epigenome-wide data collected using the Ilumina 450K or EPIC BeadChip and any pregnancy, neonatal or childhood data can join PACE. There are no restrictions on sample size, geography or ethnicity of participants and members of the consortium can propose and lead projects (https://www.niehs.nih.gov/research/atniehs/labs/epi/pi/genetics/pace/index.cfm). To date, the collaboration has largely looked at multivariable observational association, although one study (of maternal BMI) included a parental negative control study [17].

MR is increasingly being adopted to strengthen causal inference in epigenetic studies [38, 39]. The two-step MR framework is relevant to the exploration of the causal pathways linking GDM to offspring outcomes via epigenetic mechanisms [15]. The feasibility of applying MR to address questions pertaining to the potential long-term consequences of in utero exposures, such as GDM, is improving due to the increasing availability of relevant genome-wide genetic data and the development of relevant statistical methods [40–43]. However, it may not be feasible to use MR to determine a specific intrauterine effect of exposure to maternal GDM on offspring type 2 diabetes given the overlapping pathophysiology and genetic correlates between GDM and type 2 diabetes [15]. It may also not be feasible to use MR if there are no strong genetic instruments available for a particular epigenetic mark. However, genome-wide association studies of DNA methylation have been published [44, 45], with a large-scale meta-analysis underway by the Genetics of DNA Methylation Consortium (GoDMC). These efforts are generating an extensive catalogue of SNPs that tag methylation variation (meQTL) and these in turn can be used in MR and have been applied in a systematic way across many outcomes simultaneously [45].

Assessment of epigenetic measures for prediction of GDM was rare among studies identified (*n* = 4). The ability of epigenetic marks to integrate genetic and non-genetic factors in a biologically stable and technically reproducible way means they have high potential as biomarkers. Perhaps uniquely, epigenetic marks can ‘capture’ information on endogenous and exogenous exposures, including risk factors and very early consequences of disease processes, thus promising to be an effective tool in early detection and future prediction and prognosis. The use of epigenetic biomarkers in this way is established in cancer research [46], and this is an area of research that is likely to see considerably more attention in other areas in future years (including pregnancy complications, where accurate risk prediction remains poor).

In conclusion, determining whether GDM causes (via intrauterine mechanisms) increased risk of future offspring type 2 diabetes is important, because if it does there could be an intergenerational cycling or risk that would accelerate the increasing risk of type 2 diabetes and GDM. If there is a causal effect, epigenetic mechanisms could provide a potential modifiable target for intervention development to break this cycle. There is increasing optimism that epigenetic information can be used as a biomarker to predict future likelihood of adverse outcomes. As methods for identifying risk of GDM early in pregnancy (before fetal overgrowth begins) are lacking, this could be valuable for identifying women who might benefit most from more-intensive antenatal monitoring and interventions (lifestyle or pharmaceutical) to optimise fetal growth and minimise maternal hyperglycaemia. The assembled published evidence suggests that this is a fruitful avenue of research to explore. However, larger, more robust studies are required to strengthen the current evidence base.

## Electronic supplementary material

ESM 1 (PPTX 800 kb)

ESM 2 (XLSX 25.6 kb)

## Abbreviations

GDM: Gestational diabetes
miRNA: microRNA
MR: Mendelian randomisation
