# Supplementary material for: Epigenetics and gestational diabetes: a review of epigenetic epidemiology studies and their use to explore epigenetic mediation and improve prediction
Source: Diabetologia. 2019 Oct 17;62(12):2171–8. doi: 10.1007/s00125-019-05011-8 (PMC6861541; doi:10.1007/s00125-019-05011-8)
Supplement: Supplementary file 1 — (PPTX 800 kb) [file 125_2019_5011_MOESM1_ESM.pptx]

## Slide 1
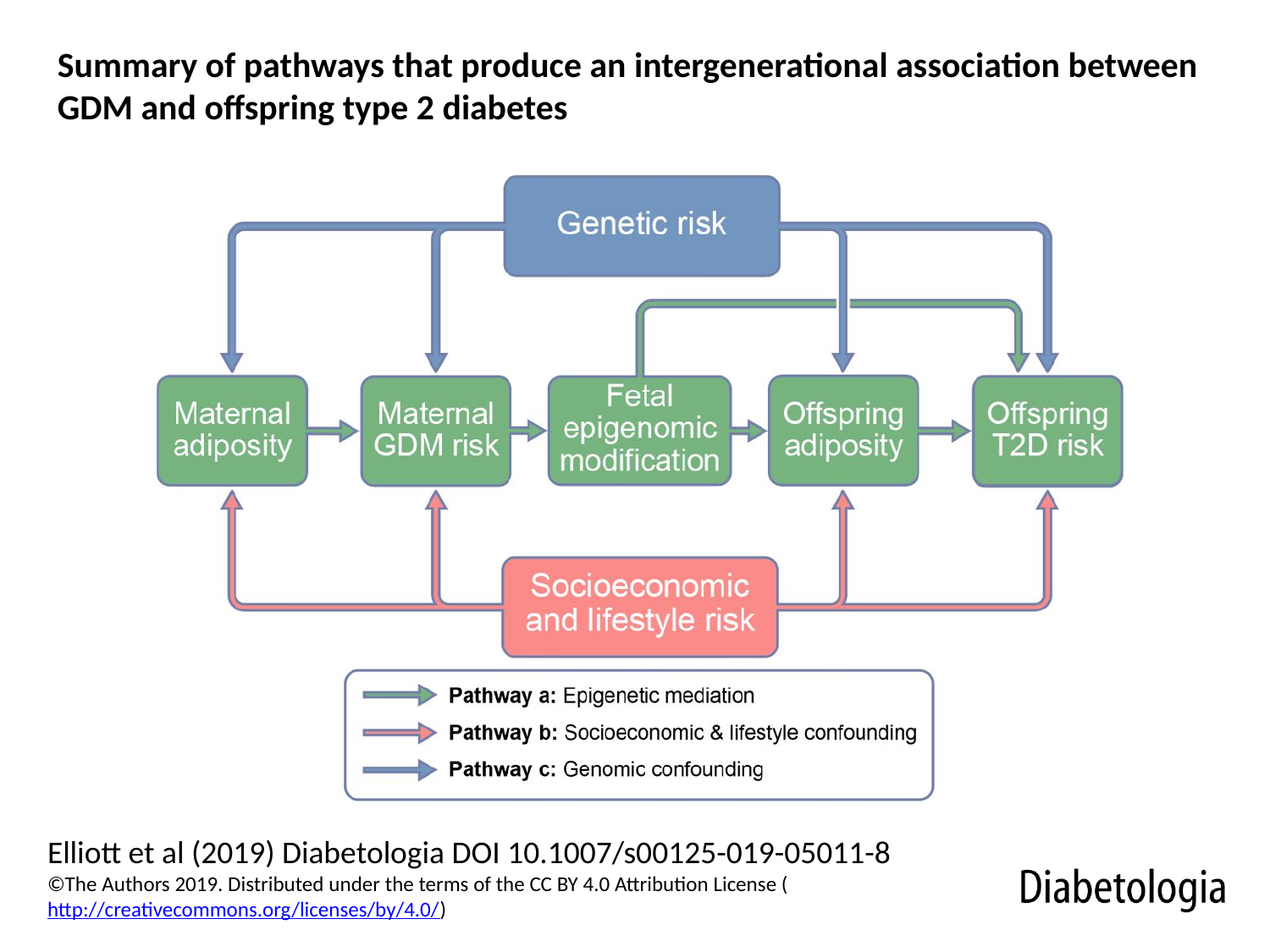

Summary of pathways that produce an intergenerational association between GDM and offspring type 2 diabetes
Elliott et al (2019) Diabetologia DOI 10.1007/s00125-019-05011-8
©The Authors 2019. Distributed under the terms of the CC BY 4.0 Attribution License (http://creativecommons.org/licenses/by/4.0/)

## Slide 2
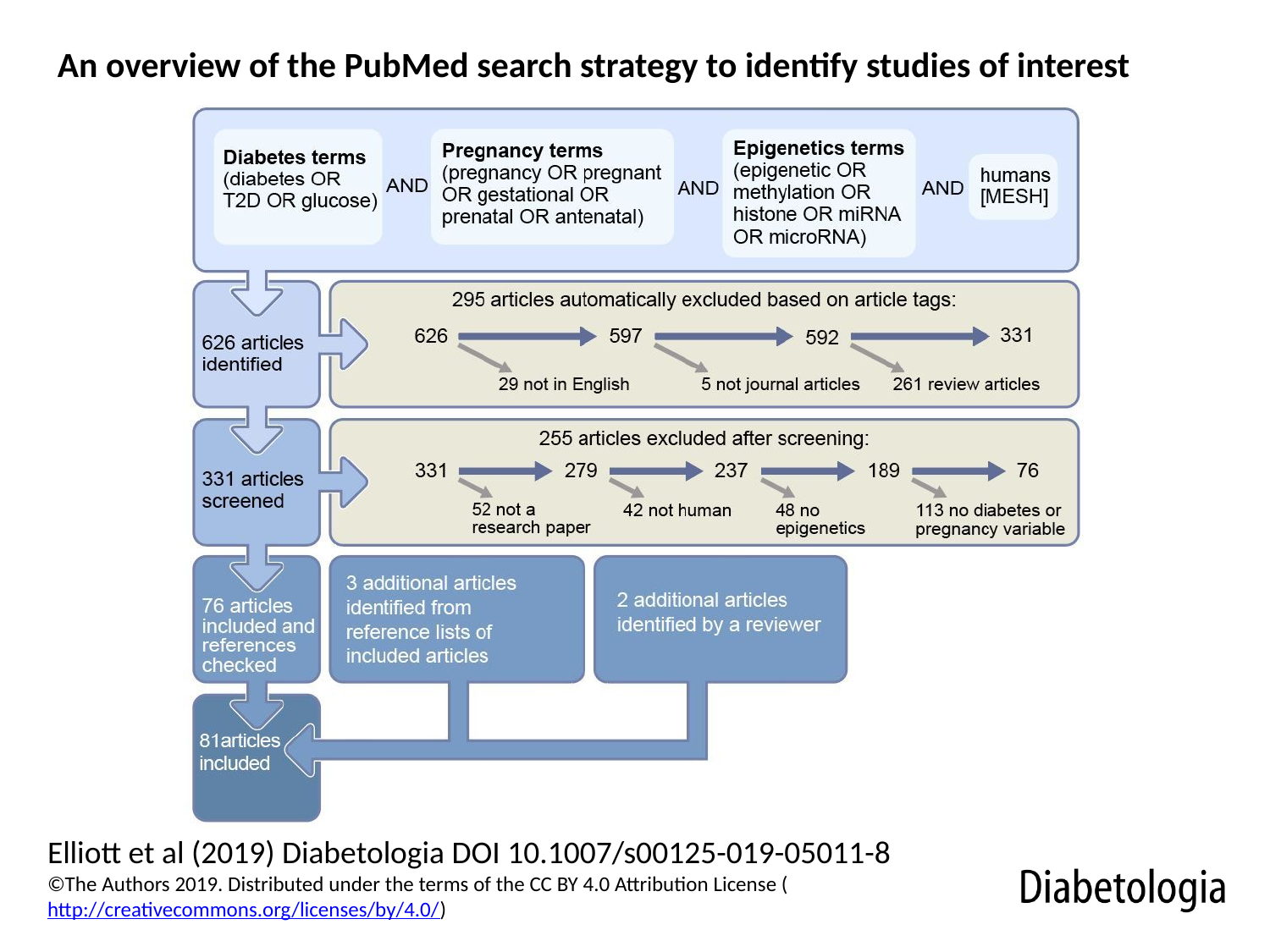

An overview of the PubMed search strategy to identify studies of interest
Elliott et al (2019) Diabetologia DOI 10.1007/s00125-019-05011-8
©The Authors 2019. Distributed under the terms of the CC BY 4.0 Attribution License (http://creativecommons.org/licenses/by/4.0/)

## Slide 3
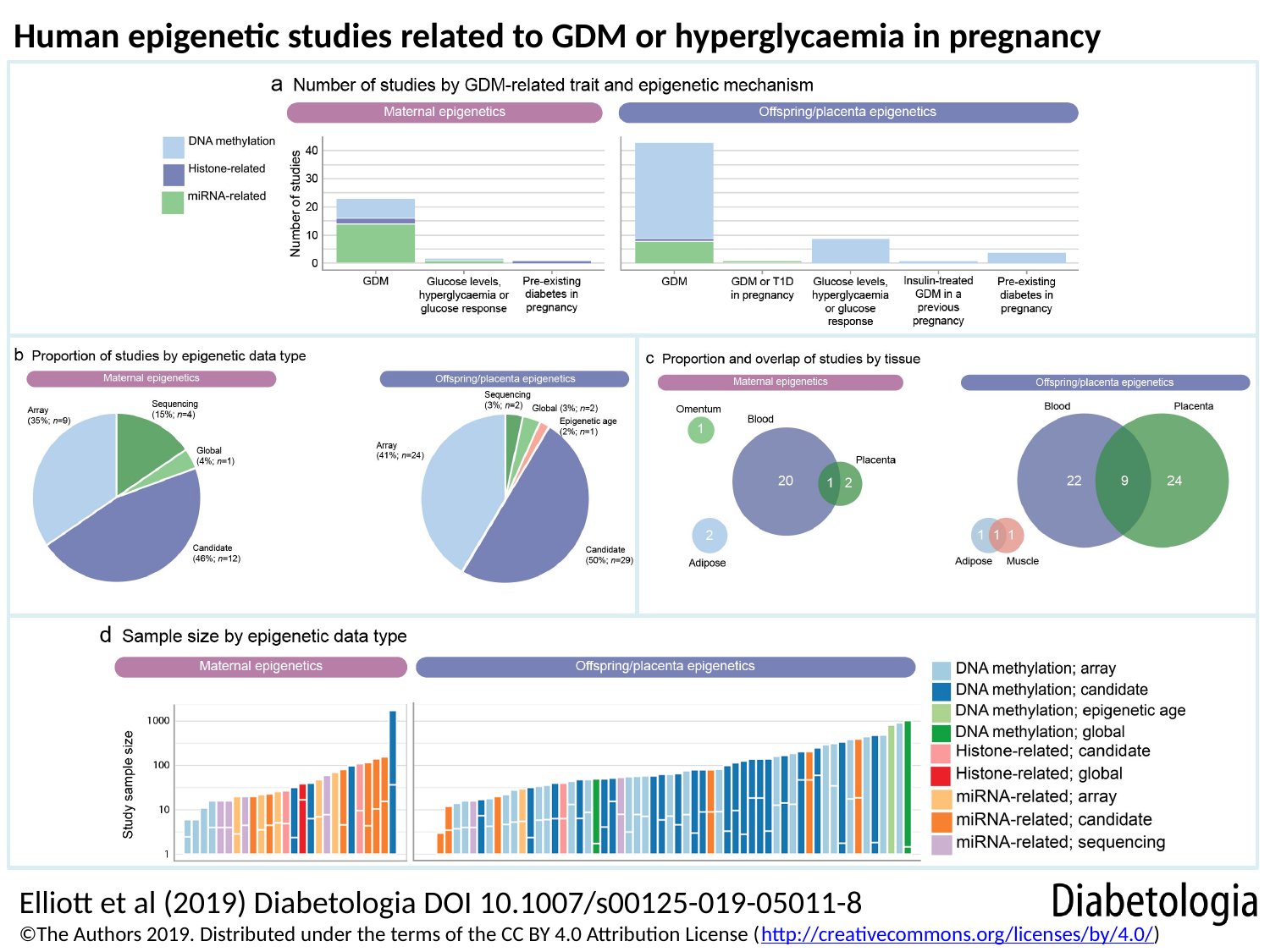

Human epigenetic studies related to GDM or hyperglycaemia in pregnancy
Elliott et al (2019) Diabetologia DOI 10.1007/s00125-019-05011-8
©The Authors 2019. Distributed under the terms of the CC BY 4.0 Attribution License (http://creativecommons.org/licenses/by/4.0/)
